# Supplementary material for: Echocardiographic view and feature selection for the estimation of the response to CRT
Source: PLoS One. 2021 Jun 10;16(6):e0252857. doi: 10.1371/journal.pone.0252857 (PMC8191962; doi:10.1371/journal.pone.0252857)
Supplement: Attachment — Submitted filename: Response to Reviewers.docx [file pone.0252857.s002.docx]

Journal Requirements:

When submitting your revision, we need you to address these additional requirements.

1. Please ensure that your manuscript meets PLOS ONE's style requirements, including those for file naming. The PLOS ONE style templates can be found at

<https://journals.plos.org/plosone/s/file?id=wjVg/PLOSOne_formatting_sample_main_body.pdf> and

<https://journals.plos.org/plosone/s/file?id=ba62/PLOSOne_formatting_sample_title_authors_affiliations.pdf>

The manuscript has been verified to meet PLOS ONE’s style requirements.

2. Please review your reference list to ensure that it is complete and correct. If you have cited papers that have been retracted, please include the rationale for doing so in the manuscript text, or remove these references and replace them with relevant current references. Any changes to the reference list should be mentioned in the rebuttal letter that accompanies your revised manuscript. If you need to cite a retracted article, indicate the article’s retracted status in the References list and also include a citation and full reference for the retraction notice.

All references have been reviewed with no modification.

3. Thank you for including your ethics statement:

“ The study was reviewed by an independent ethics committee (regional ethic committee validation number: 35RC14-9767) and conducted in accordance with the ‘Good Clinical Practice’ Guidelines in the Declaration of Helsinki. All patients provided written informed consent. “

Please amend your current ethics statement to include the full name of the ethics committee/institutional review board(s) that approved your specific study.
Once you have amended this/these statement(s) in the Methods section of the manuscript, please add the same text to the “Ethics Statement” field of the submission form (via “Edit Submission”).
For additional information about PLOS ONE ethical requirements for human subjects research, please refer to <http://journals.plos.org/plosone/s/submission-guidelines#loc-human-subjects-research>.

The ethics statement has been added to the manuscript.

4. Please include captions for your Supporting Information files at the end of your manuscript, and update any in-text citations to match accordingly. Please see our Supporting Information guidelines for more information: http://journals.plos.org/plosone/s/supporting-information.

The caption for the Supporting Information has been added at the end of the manuscript.

**Comments to the Author**

1. Is the manuscript technically sound, and do the data support the conclusions?

The manuscript must describe a technically sound piece of scientific research with data that supports the conclusions. Experiments must have been conducted rigorously, with appropriate controls, replication, and sample sizes. The conclusions must be drawn appropriately based on the data presented.

Reviewer #1: Yes

Reviewer #2: Yes

2. Has the statistical analysis been performed appropriately and rigorously?

Reviewer #1: Yes

Reviewer #2: Yes

3. Have the authors made all data underlying the findings in their manuscript fully available?

The [PLOS Data policy](http://www.plosone.org/static/policies.action#sharing) requires authors to make all data underlying the findings described in their manuscript fully available without restriction, with rare exception (please refer to the Data Availability Statement in the manuscript PDF file). The data should be provided as part of the manuscript or its supporting information, or deposited to a public repository. For example, in addition to summary statistics, the data points behind means, medians and variance measures should be available. If there are restrictions on publicly sharing data—e.g. participant privacy or use of data from a third party—those must be specified.

Reviewer #1: Yes

Reviewer #2: Yes

4. Is the manuscript presented in an intelligible fashion and written in standard English?

PLOS ONE does not copyedit accepted manuscripts, so the language in submitted articles must be clear, correct, and unambiguous. Any typographical or grammatical errors should be corrected at revision, so please note any specific errors here.

Reviewer #1: Yes

Reviewer #2: Yes

5. Review Comments to the Author

Please use the space provided to explain your answers to the questions above. You may also include additional comments for the author, including concerns about dual publication, research ethics, or publication ethics. (Please upload your review as an attachment if it exceeds 20,000 characters)

Reviewer #1: The article is original, well written. It analyzes the use of strain in the prediction of CRT responders.
Over time, different methods and different echocardiographic criteria have been used in predicting the response to CRT. In the introduction the authors may cite the review "Non-responders to cardiac resynchronization therapy: Insights from multimodality imaging and electrocardiography. A brief review" published in International Journal of Cardiology (doi: 10.1016 / j.ijcard.2016.09.037) and other articles related to old and new echocardiographic methods useful in identifying patient responders to CRT.

Thank you for your review and for this comment. References have been added to emphasize the recent echocardiographic methods of the literature, including the pertinent citation mentioned by the reviewer.

Reviewer #2: The authors investigated the relative importance of strain-based features obtained from the 4ch, 3ch and 2ch apical views in identifying patients responders to cardiac resynchronization therapy. They found 20 features as the most important, 60% of them obtained from the 4ch apical view. The study is interesting, well written and conducted. However I have some comments:

1) How do you translate your findings in routine clinical practice? You identified 20 features as the most important, however some of them, particularly those derived from integrals, seem not easy to obtain and/or time-consuming, thus their application in clinical practice seem doubtful. Please comment it.

We understand that the nature of integrals may be considered as complex. However, the process of obtaining these markers is completely automatic once the application of a standard cardiac strain study has been performed. Indeed, the only manual task for a standard cardiac strain study is the segmentation of the LV. In our method, we also require a manual verification of the instant of aortic valve closure, which is classically performed in echo analyses. The generation of strain curves and the calculation of the strain features are performed in a completely automatic fashion. This aspect increases the reproducibility of the results and allow for an easy translation to the clinics. We have clarified this point in the manuscript.

2) Figure 2 should be improved. The text in the figure is not legible.

We are sorry Figure 2 is not legible in the manuscript. However, the figure is legible in the tiff file by downloading it with the link accessible in the top right corner of Figure 2 in the manuscript.

3) Table 3 is a little bit confusing. Please improve it.

We have added a new column in Table 3 depicting the Rank of each group of features. We have also changed the legend of this Table. We believe that these modifications should avoid confusions while reading this table.

6. PLOS authors have the option to publish the peer review history of their article ([what does this mean?](https://journals.plos.org/plosone/s/editorial-and-peer-review-process#loc-peer-review-history)). If published, this will include your full peer review and any attached files.


If you choose “no”, your identity will remain anonymous but your review may still be made public.


**Do you want your identity to be public for this peer review?** For information about this choice, including consent withdrawal, please see our [Privacy Policy](https://www.plos.org/privacy-policy).

Reviewer #1: No

Reviewer #2: No

[NOTE: If reviewer comments were submitted as an attachment file, they will be attached to this email and accessible via the submission site. Please log into your account, locate the manuscript record, and check for the action link "View Attachments". If this link does not appear, there are no attachment files.]

While revising your submission, please upload your figure files to the Preflight Analysis and Conversion Engine (PACE) digital diagnostic tool, <https://pacev2.apexcovantage.com/>. PACE helps ensure that figures meet PLOS requirements. To use PACE, you must first register as a user. Registration is free. Then, login and navigate to the UPLOAD tab, where you will find detailed instructions on how to use the tool. If you encounter any issues or have any questions when using PACE, please email PLOS at [figures@plos.org](mailto:figures@plos.org). Please note that Supporting Information files do not need this step.
